# NEIGHBORHOOD SOCIOECONOMIC POSITION, LIVING ARRANGEMENTS, AND CARDIOMETABOLIC DISEASE AMONG OLDER PUERTO RICANS

**DOI:** 10.1093/geroni/igad104.0846

**Published:** 2023-12-21

**Authors:** Nekehia Tamara Quashie, Catherine Garcia, Gabriella Meltzer, Flavia Andrade, Amilcar Matos-Moreno

**Affiliations:** University of Rhode Island, Kingston, Rhode Island, United States; Syracuse University, Syracuse, New York, United States; Columbia University Mailman School of Public Health, New York City, New York, United States; University of Illinois--Urbana-Champaign, Urbana, Illinois, United States; Pennsylvania State University, State College, Pennsylvania, United States

## Abstract

Cardiometabolic diseases are among the leading causes of mortality worldwide and are increasingly prevalent in rapidly aging populations such as Puerto Rico. Neighborhood socioeconomic position (SEP) and living arrangements are increasingly recognized as important determinants of cardiometabolic health but have not been examined within Puerto Rico. Using longitudinal data from the Puerto Rican Elderly Health Conditions Project (Waves I 2002/03, and II 2006/07), linked with 2000 Census data for neighborhood-level conditions, this study examined the association between neighborhood SEP, living arrangements, and risk of cardiometabolic conditions among island-dwelling Puerto Ricans aged 60 and older, who lived in the same residence across both waves (N=2,769). Findings show that residence in a socioeconomically advantaged neighborhood was positively associated with reporting having one cardiometabolic condition at baseline, but neighborhood SEP was unrelated to developing cardiometabolic conditions at follow-up. Living without a partner - alone, with children, or with others - was negatively associated with reporting having and developing cardiometabolic conditions relative to living with a partner. Living arrangements significantly modified the relationship between neighborhood SEP and cardiometabolic conditions. Compared to living with a partner, living alone was associated with lower risk of reporting having one condition, and living with children was associated with lower risk of developing one cardiometabolic condition when living within a socioeconomically advantaged neighborhood. Living arrangements are more salient to cardiometabolic health than neighborhood SEP. Social services focused on household composition and familial support are needed to identify older Puerto Ricans potentially at risk of underdiagnosed chronic conditions.

